# Clusters of Injuries From Motorcycle Collisions: Exploratory Factor Analysis of a Single Institution Trauma Registry

**DOI:** 10.7759/cureus.18713

**Published:** 2021-10-12

**Authors:** Kelsey A Rankin, Adam M Lukasiewicz, Maia Ou, Theodore Zaki, David Molho, Yasmmyn Salinas, Alex Goel, Michael P Leslie, Daniel H Wiznia

**Affiliations:** 1 Department of Orthopaedics, Yale New Haven Hospital, New Haven, USA; 2 Department of Psychiatry, Zuker School of Medicine at Hofstra, Hempstead, USA; 3 Department of Dermatology, Yale New Haven Hospital, New Haven, USA; 4 Department of Chronic Disease, Epidemiology, Yale New Haven Hospital, New Haven, USA; 5 Department of Otolaryngology, Head and Neck Surgery, Mount Sinai Hospital, New York, USA

**Keywords:** motorcycle collision, factor analysis, exploratory factor analysis, injury cluster, trauma

## Abstract

Objective

With the goal of guiding acute management of associated injuries motorcycle trauma patients, this study aims to identify patterns of associated injuries after motorcycle collisions using exploratory factor analysis.

Methods

We conducted a retrospective review at a Level 1 trauma center of all patients who presented after motorcycle collisions resulting in trauma system activations between July 2, 2002 and December 31, 2013. We performed exploratory factor analysis on this dataset to identify sets of injuries that cluster together.

Results

We identified 1,050 patients who presented for trauma after a motorcycle collision. These patients had 3,101 injuries, including 1,694 fractures. Using exploratory factor analysis, we developed a model with four latent factors that explained approximately half of the variance in injuries. These factors were defined by: head and cervical spine injuries; extremity injuries; abdomen, pelvis and upper extremity injuries; and shoulder girdle and thorax injuries. We also found a novel injury pattern relationship between forearm shaft/wrist and lower extremity injuries.

Conclusions

Motorcycle trauma results in distinct clusters of associated injuries likely due to common motorcycle collision patterns, most notably a novel relationship between forearm shaft/wrist and lower extremity injuries that merits further exploration, and could play a role during secondary survey.

## Introduction

Patients involved in motorcycle collisions often have major injuries to more than one region or organ system [1]. Moreover, the presence of severe injuries in one organ system may predict the presence of severe injuries in other organ systems [2]. Understanding the associations between these injuries is important for clinical care, research, and the development of strategies for injury mitigation.

Previous studies have identified injuries that are commonly associated with each other after motorcycle trauma, such as pelvic fractures coinciding with intra-abdominal injuries [3], and femur fractures coinciding with tibia fractures [4]. While the knowledge of these particular pair-wise associations is helpful, there are many potential combinations of connected injuries, making the process of both discovering the associations and using them in clinical practice challenging. However, there remains a clear gap in our knowledge of what injuries cluster together following motorcycle collisions. This knowledge could be used to aid in acute management of injuries, understand the underlying common mechanism of injuries causing the clusters, and ultimately create guidance on necessary protective measures. We therefore sought to determine if there are broader, overarching patterns of association between injuries related to motorcycle collisions.

## Materials and methods

We conducted a retrospective review of a motorcycle trauma registry at our institution within the time frame of July 2, 2002 to December 31, 2013. Our institution is a regional Level 1 Trauma Center adjacent to a major highway system. The database included all patients presenting to our institution after a motorcycle collision who were evaluated by the general surgery trauma team, had injuries resulting in trauma team activation, and/or had documented traumatic injuries based on standardized coding.

Our motorcycle trauma registry includes a wide variety of patient demographic, medical, and injury characteristics, as well as data about the hospital course. Injury Severity Score (ISS) was computed using the 2008 update of Abbreviated Injury Scale [5] by an orthopaedic surgery resident trained in the management of trauma and familiar with the scale. Fractures were recorded by Orthopaedic Trauma Association/AO Foundation classification [6]. Other fracture-related characteristics including open fracture, associated compartment syndrome, and requirement for soft tissue coverage were collected. Non-orthopaedic injuries were included in the registry, and were grouped as: closed head injuries, chest injuries, and abdominal injuries. A senior orthopedic surgery resident reviewed all patient charts and abstracted the data. The data were checked by three medical students, and any discrepancies were resolved by the senior author.

Patient and injury characteristics, treatment, and outcomes were tabulated. Exploratory Factor Analysis (EFA) was performed to look for underlying structure in the injury data. EFA is a well-described technique to attempt to simplify data structures, thereby making the underlying relationships more apparent [7]. It has been used in clinical analyses as a reduction tool to understand correlations of variables, to determine relationships between clinical characteristics of pain, to assess for indicators and determinants of asthma, and to validate the factorial structure of existing measures [8-15]. Conceptually, EFA generates a model in which there are unobservable latent factors that explain, in part, the observed data. These latent factors are constructed by identifying data elements which are highly correlated, based on the assumption that the correlation between these variables is due to the effect of the latent variable. The authenticity of these results depends on the reliability and accuracy of the assumptions made, and thus were validated by a PhD in statistics to ensure no erroneous assumptions were made. Many different models with varying numbers of factors can be developed with EFA, so model selection is a vital aspect of the process.

The Kaiser-Meyer-Olkin measure of sampling adequacy was 0.607, indicating mediocre sampling adequacy by convention. Bartlett’s test of sphericity had p<0.0001, indicating adequate partial correlation of the data for factor extraction. In this case, the tetrachoric covariance matrix was used. Factors were extracted using principal axis factoring [14]. The scree plot method was used to visually determine the appropriate number of factors to be retained [16]. An orthogonal varimax rotation was performed prior to interpretation of the factors. The varimax rotation used in this study makes an assumption that the factors are uncorrelated. Oblique rotations do not make the same assumption about the data structure, but they are less straightforward to interpret. The same factor analysis with an oblique rotation did not substantially change the overall factor structure, so we chose the orthogonal rotation for ease of interpretation. Factor loadings were considered to be appropriate for defining factors when the magnitude was 0.4 or more [17].

For demographic and injury data, missing data were reported. For EFA, patients with missing data for a covariate investigated in the factor analysis were excluded from the analysis. In total, 4.9% of patients were missing data and excluded from EFA. All statistical analyses were performed using Stata 13.1 (StataCorp, College Station, TX).

We chose to use EFA to attempt to identify these overarching patterns. EFA is a statistical method to discover unobserved or latent factors which can explain the observed patterns in the data [18]. The method uses the covariance between the observed variables to construct the latent factors. This technique has been used primarily in social science and psychological research [19].

This study was approved by the Yale University Human Research Protection Program HIC#1403013641. Written informed consent was obtained from all research subjects during their initial hospitalization period.

## Results

A total of 1,050 patients were identified in our motorcycle trauma database, with a total of 3,101 injuries, including 1,694 fractures. Notably, the cohort had a wide age range, was predominantly male (88.8%), and unhelmeted (68.9%) (Table 1; Table A1). Notably, in-hospital mortality was approximately 6%, and almost one-third of patients required intensive care (Table 2). We observed a wide range of bodily injuries to different organ systems (Tables 3, 4).

**Table 1:** Patient characteristics.

Overall	1,050	
Age		
<19	66	6.3%
19-29	313	29.8%
30-39	203	19.4%
40-49	244	23.3%
> 50	217	20.6%
Male sex	927	88.8%
Helmeted	326	31.1%
Injury Severity Score		
1-9	403	38.4%
10-15	260	24.8%
16-20	121	11.5%
> 20	257	24.5%

**Table 2:** Hospital course.

Overall	1,050	
Death	63	6.1%
ICU stay	326	29.6%
Length of stay (days)		
1	269	25.6%
2	136	13.0%
3	106	10.1%
4-5	137	13.0%
6-20	293	27.9%
21+	103	9.8%
Unknown	6	0.6%
Disposition		
Home	689	23.5%
Skilled nursing facility	247	65.6%
Other	107	10.2%
Unknown	7	0.7%

**Table 3:** Extremity and pelvic injuries. OTA/AO: Orthopaedic Trauma Association/AO Foundation.

OTA/AO Classification	Fractures	Open	
Upper extremity			
11	20	0	0.0%
12	18	6	33.3%
13	15	5	33.3%
14	99	2	2.0%
15	141	3	2.1%
21	25	7	28.0%
22	45	19	42.2%
23	74	10	13.5%
7	144	14	9.7%
Lower extremity			
31	36	3	8.3%
32	72	24	33.3%
33	40	19	47.5%
34	28	17	60.7%
41	91	26	28.6%
42	80	44	55.0%
43	73	40	54.8%
44	89	25	28.1%
8	143	48	33.6%
Pelvis			
61	109	8	7.3%
62	55	2	3.6%
Total	1397	322	23.0%

**Table 4:** Axial and non-orthopedic injuries.

Spine fracture	233	22.2%
Cervical	94	5.5%
Thoracic	106	6.3%
Lumbar	97	5.7%
Skull fracture	239	22.8%
Intracranial injury	372	35.4%
Facial fracture	171	16.3%
Chest injury	360	34.3%
Abdominal injury	143	13.6%

**Figure 1:**
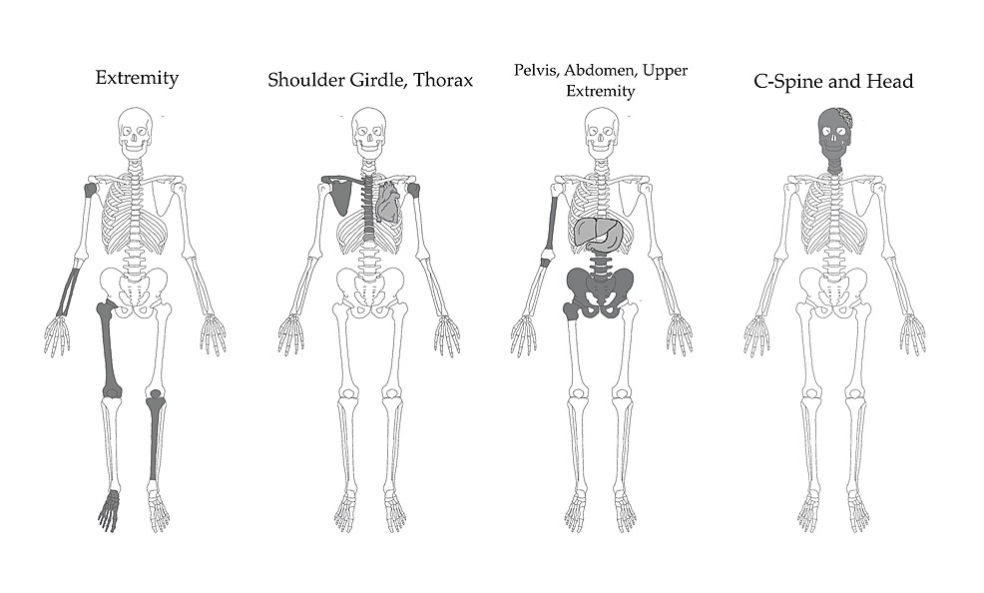
Injury patterns based on factor loadings.

The best-fit latent factor model included four factors (Table 5). The four-factor solution accounted for 44% of the variance in the occurrence of injuries. The injuries that load onto these four factors are depicted graphically in Figure 1. One factor was defined by extremity injuries. Another factor was defined by injuries to the thorax, shoulder girdle, and thoracic spine. The third factor was defined by injuries to the acetabulum and bony pelvis, lumbar spine, abdomen, proximal radius and ulna, and humeral shaft. The final factor was defined by injuries to the head and cervical spine, and negative loading of foot and lateral malleolus fractures. The full factor loading and communality for each variable are shown in Table A2. Injuries to the foot cross-loaded onto the extremity factor and the head and cervical spine factor, although the loading was negative for the head and cervical spine factor. Injuries to the proximal humerus cross-loaded onto the extremity factor and the shoulder girdle and thorax factor, and injuries to the hip cross-loaded on the extremity factor and the abdomen, pelvis, and upper extremity factor.

**Table 5:** Latent factors. Injury code corresponds with concomitant numerical coding in Table A2. Injuries are reported according to Orthopaedic Trauma Association/AO Foundation system where possible.

Factor	Injury code	Prevalence of multiple defining injuries	Variance explained
Extremity	Positive: 11, 22, 23, 31, 32, 33, 34, 41, 42, 8 Negative: 13	39.7%	15.0%
Shoulder girdle and thorax	Positive: 11, 14, 15, 52, Chest Injury	44.1%	12.4%
Pelvis, abdomen, and upper extremity	Positive: 12, 21, 31, 53, 61, 62, Abdominal Injury	38.5%	9.9%
Cervical spine and head	Positive: 51, 91, 92, Intracranial Injury Negative: 44, 8	53.4%	8.7%

## Discussion

We identified four clusters of injuries that occur in motorcycle collision patients using EFA based on a retrospective review of our institution’s motorcycle trauma registry. These clusters of injuries largely correspond with anatomic relationships and make intuitive sense in relation to common motorcycle collision mechanisms of injury.

We were not able to assess the relationships between the clusters we identified and the mechanism of injury due to a lack of sufficient collision data in the medical records. This is an area that we believe merits further follow-up in an additional study. However, based on the classification of motorcycle crash mechanisms [20], we suspect that the head and cervical spine factor corresponds with head-leading collisions. Similarly, we suspect that the abdomen, pelvis, and upper extremity factor corresponds with fuel tank. The shoulder girdle and thorax factor may correspond with direct vertical impact injuries, and the extremity factor may correspond with lowside injuries. The possible links between these injury clusters and the mechanisms of motorcycle trauma need to be further investigated. Understanding these links between mechanism and clinical injury may help inform efforts to improve rider and motorcycle safety, and reduce the burden of motorcycle-related trauma. In addition, these mechanisms of injury could serve to influence acute triage and potential guidelines for protective measures while riding a motorcycle.

These clusters also demonstrate relationships that have been previously shown in the literature. There is a known association between pelvis fractures and femur fractures [4], and pelvis fractures are commonly associated with intra-abdominal injuries [3]. In our study, patients who had at least one defining cervical spine or head injury had a 53.4% chance of having another injury within the same factor. This is in line with the findings of Kraus et al. who demonstrated that the odds that a patient has a traumatic brain injury were 3.5 times greater if the patient presented with facial injury and 6.5 times greater with facial fractures [21]. We hope that our results may be beneficial to the clinical management of injured motorcyclists, particularly in the efficient triage and work-up of these patients based on knowledge of which injuries tend to cluster together. We recommend that when a patient presents with facial injury, the trauma team should also evaluate the patient for C-spine and head trauma, and patients who present with shoulder girdle injuries should be evaluated for thorax injuries.

Some of these injury clusters we identified have not been previously reported. We have not found an association in the literature between forearm shaft/wrist injuries specifically and lower extremity injuries in the motorcycle trauma population. Similarly, we are not aware of any reported association between humeral shaft and proximal radial/ulna injuries with injuries to the hip/pelvis, abdomen, and lumbar spine. These associations should be examined directly in future work with a different dataset.

Our registry only included patients who required hospital care, and it does not include patients who succumbed to their injuries at the scene of the accident. Patients were included in the registry based on the chart documentation of the mechanism and nature of their injuries. Inaccuracies in documentation may have led to some motorcycle trauma patients not being identified. Additionally, follow-up was limited to the initial hospitalization. The four factors identified in our study accounted for about 45% of the variation in injuries. This value is lower than ideal values for total variance explained in the social sciences, but in the range of typical values for EFA studies included in a meta-analysis [22]. The lower total variance may be in part due to the heterogeneity and randomness in collision injuries. While EFA is a useful technique for investigating structure in data, this technique is by definition exploratory. The factors identified here are sensible and are largely consistent with previously published work. Confirmatory factor analysis, ideally with an entirely distinct dataset, will be needed to check the validity and reliability of these clusters.

In our model, tibial plafond injuries and hand injuries had low communality and did not load appreciably onto any factor. The variations in these injuries are not well explained by the four latent factors we identify. Three variables had significant cross-loading: hip, proximal humerus, and foot injuries. In this case, there is no a priori need for construct purity, and cross-loading does not invalidate the model. The cross-loading of the proximal humerus and hip injuries could reflect that these anatomic areas are the transition points between the appendicular and axial skeleton, and therefore are associated with injuries in each cluster.

## Conclusions

Based on EFA we identified four clusters of injuries following motorcycle collisions. Some of these reinforced previously established motorcycle collision mechanisms, however, some relationships were novel, including the relationship between forearm shaft/wrist and lower extremity injuries, and merit further exploration. We believe these relationships should aid in guiding the acute triage of patients following motorcycle collision, and can serve as a springboard for future research to drive guidelines on necessary protective measures.

## Appendices

**Table 6:** A1. Patient characteristics.

Overall	1,050	
Age		
<19	66	6.3%
19-24	165	15.7%
25-29	148	14.1%
30-34	95	9.1%
35-39	108	10.3%
40-44	114	10.9%
45-49	130	12.4%
50-54	98	9.3%
55-59	60	5.7%
> 60	59	5.6%
Unknown	7	0.7%
Male sex	927	88.8%
Race/Ethnicity		
Black	128	12.3%
Hispanic	116	11.1%
White	776	74.3%
Other/Unknown	24	2.3%
Helmeted	326	31.1%
Insurance		
Private	550	52.4%
Medicaid	336	32.0%
Medicare	52	5.0%
Worker's comp	7	0.7%
Other	98	9.3%
Unknown	7	0.7%
Toxicology		
Alcohol only	207	20.6%
Controlled Substances	116	11.5%
Negative	682	67.9%
Injury Severity Score		
1-4	129	12.3%
5-9	274	26.1%
10-15	260	24.8%
16-20	121	11.5%
21-30	176	16.8%
>30	81	7.7%
Unknown	9	0.9%
GCS on Presentation		
15	742	70.7%
12-14	92	8.8%
4-11	24	2.3%
3	97	9.2%
Unknown	95	9.0%
Loss of Consciousness		
Yes	351	33.6%
No	459	44.0%
Unknown	233	22.3%

**Table 7:** A2. Exploratory factor analysis loadings and communality.

Injury	Factor Loadings				Communality
	Factor 1	Factor 2	Factor 3	Factor 4	
Proximal Humerus (11)	0.5669	0.5250	-0.1310	-0.0137	0.6143
Diaphyseal Humerus (12)	0.0515	-0.0472	0.6026	0.3788	0.5115
Distal Humerus (13)	-0.8489	-0.0683	0.176	0.0370	0.7577
Scapula (14)	-0.0156	0.7589	-0.1387	0.0081	0.5955
Clavicle (15)	0.1183	0.6162	-0.3687	0.1199	0.544
Proximal Forearm (21)	0.1781	-0.1693	0.4545	0.0525	0.2697
Diaphyseal Forearm (22)	0.5082	-0.1542	0.3805	0.3894	0.5785
Distal Forearm (23)	0.4802	-0.1471	0.1016	0.2452	0.3227
Proximal Femur (31)	0.4685	0.1405	0.4095	-0.2390	0.4641
Diaphyseal Femur (32)	0.6328	-0.0604	0.2312	0.1344	0.4756
Distal Femur (33)	0.5132	-0.0743	0.3386	-0.0817	0.3903
Patella (34)	0.6276	-0.0833	-0.0625	-0.0964	0.414
Proximal Leg (41)	0.6091	-0.1601	0.0506	-0.1128	0.4119
Diaphyseal Leg (42)	0.5406	-0.1549	-0.0162	-0.3530	0.4412
Distal Leg (43)	0.0111	-0.3889	0.1579	-0.3228	0.2805
Malleoli (44)	0.0857	-0.0834	0.1860	-0.4187	0.2242
Cervical Spine (51)	-0.1448	0.3926	0.0464	0.4096	0.345
Thoracic Spine (52)	-0.0313	0.6401	0.3131	0.1699	0.5376
Lumbar Spine (53)	-0.0996	0.3048	0.5130	-0.0994	0.3759
Pelvic Ring (61)	-0.0951	-0.0077	0.7633	0.0113	0.5919
Acetabulum (62)	0.0518	0.0923	0.6607	-0.1824	0.481
Hand (7)	0.0713	-0.2017	0.0457	0.2169	0.0949
Foot (8)	0.4401	0.0611	-0.1763	-0.444	0.4256
Cranium (91)	-0.0146	0.0922	-0.0266	0.8162	0.6755
Maxillofacial Bones (92)	-0.0158	0.0880	0.0225	0.7192	0.5258
Intracranial	-0.0880	0.0846	-0.0781	0.6128	0.3966
Chest	-0.0964	0.8078	0.2454	0.0506	0.7246
Abdominal	-0.1156	0.3699	0.4967	-0.0304	0.3978
